# Pharmacological and Benefit-Risk Profile of Once-Weekly Basal Insulin Administration (Icodec): Addressing Patients’ Unmet Needs and Exploring Future Applications

**DOI:** 10.3390/jcm13072113

**Published:** 2024-04-05

**Authors:** Ylenia Ingrasciotta, Giacomo Vitturi, Gianluca Trifirò

**Affiliations:** 1 Diagnostic and Public Health Department, University of Verona, 37134 Verona, Italy; ylenia.ingrasciotta@univr.it (Y.I.); giacomo.vitturi@univr.it (G.V.); 2 Academic Spin-off “Innovative Solutions for Medical Prediction and Big Data Integration in Real World Setting Srl—INSPIRE SRL”, University of Messina, 98125 Messina, Italy

**Keywords:** diabetes mellitus, insulin icodec, adherence, quality of life, environmental sustainability, unmet needs, randomized clinical trial

## Abstract

Diabetes mellitus (DM) is a chronic metabolic disease affecting over 500 million people worldwide, which leads to severe complications and to millions of deaths yearly. When therapeutic goals are not reached with diet, physical activity, or non-insulin drugs, starting/adding insulin treatment is recommended by international guidelines. A novel recombinant insulin is icodec, a once-weekly insulin that successfully completed phase III trials and that has recently obtained the marketing authorization approval from the European Medicines Agency. This narrative review aims to assess icodec pharmacological and clinical features concerning evidence on benefit–risk profile, as compared to other basal insulins, addressing the potential impact on patients’ unmet needs. Icodec is a full agonist, recombinant human insulin analogue characterized by an ultra-long half-life (196 h), enabling its use in once-weekly administration. Phase III randomized clinical trials involving more than 4000 diabetic patients, mostly type 2 DM, documented non-inferiority of icodec, as compared to currently available basal insulins, in terms of estimated mean reduction of glycated hemoglobin levels; a superiority of icodec, compared to control, was confirmed in insulin-naïve patients (ONWARDS 1, 3, and 5), and in patients previously treated with basal insulin (ONWARDS 2). Icodec safety profile was comparable to the currently available basal insulins. Once-weekly icodec has the potential to improve patients’ adherence, thus positively influencing patients’ treatment satisfaction as well as quality of life, especially in type 2 DM insulin-naïve patients. An improved adherence might positively influence glycemic target achievement, reduce overall healthcare costs and overcome some of the unmet patients’ needs. Icodec has the potential to emerge as a landmark achievement in the evolution of insulin therapy, with a positive impact also for the National Health Services and the whole society.

## 1. Introduction

Diabetes mellitus (DM) is one of the most common chronic metabolic diseases, which nowadays affects more than 500 million subjects [1]. The International Diabetes Federation (IDF) estimates that this number will rise to 643 million by 2030 [1]. Diabetes can lead to life-threatening complications, such as retinopathy, nephropathy, neuropathy, and cardiovascular disease, and it is one of the major causes of death, accounting for 6.7 million deaths in 2021 alone [1,2]. Type 2 diabetes mellitus (T2DM) is the most common type of diabetes, accounting for 90% of cases [1].

The rising number of people with T2DM has an important impact, in terms of both clinical effects and economic burden, on healthcare systems, ranging from 2.5% to 15% of the total healthcare expenditure in each European Country [3].

The most recent guidelines of the American Diabetes Association (ADA) and of the European Society of Cardiology provide recommendations on prevention, diagnosis, monitoring procedure and therapeutic strategies [4,5]. Concerning the pharmacological therapy, the guidelines recommend the use of specific antidiabetic drugs based on therapeutic goals, such as weight loss, minimizing hypoglycemia risk, and preventing/managing cardiovascular or renal complications. In late-stage diabetes or when glycemic control is not achieved with non-insulin drugs, such as oral antidiabetics and GLP1-RA, the addition of or the switch to insulin therapy is recommended.

Over the past 40 years, various recombinant insulin analogues have been developed, employing distinct technologies tailored to achieve specific objectives, such as the use of rapid-release or extended-release formulations [6]. Nowadays, ongoing technological advancements are leading to the development of novel insulin formulations, which will hopefully have the potential to improve both the efficacy and safety of insulin therapy [7].

Second generation basal insulins, such as degludec and glargine 300, which are characterized by an extended duration of action and by a low pronounced pharmacokinetic peak, have shown a reduced risk of hypoglycemia while maintaining a good glycemic control, compared to other basal insulins [8,9,10]. Indeed, their use is recommended by the Italian SID–AMD and by international ADA guidelines [4,11]. In particular, insulin degludec has demonstrated a reduction in severe and symptomatic nocturnal hypoglycemia, and a reduction in insulin dose and greater flexibility, compared to insulin glargine 300 [12,13].

Unlike fast-acting insulins, which should be administered immediately before meals, basal insulins are typically taken once or twice daily, with the option to further split the dosage if necessary [14]. Recently, some novel recombinant insulin formulations, such as icodec and efsitora-alfa, have been designed for once-weekly administration. The extended-release formulation was used to provide an option to simplify the treatment for diabetic patients, drastically reducing the number of injections from once/twice per day to once per week. Specifically, efsitora-alfa completed a phase 2 program [15] and recently started phase 3 trials (QWINT trials), while icodec successfully already completed the ONWARDS phase 3a trial program, consisting of six multi-center randomized clinical trials (RCTs). These trials collectively involved over 4000 adults with type 1 (N = 580) or type 2 DM (N = 3746), and they all met their primary endpoints. Based on data from the ONWARDS clinical trial program, the manufacturer submitted a biologics license application (BLA) for once-weekly insulin icodec for the treatment of diabetes mellitus to the US Food & Drug Administration (FDA) and to the European Medicines Agency (EMA) in April and May 2023, respectively [16,17]. On 21st March 2024, the EMA issued a favourable opinion for the marketing authorization of the medicinal product Awiqli (insulin icodec) [18], manufactured by Novo Nordisk, headquartered in Bagsværd, Denmark.

This paper aims to provide an overview of the pharmacological and benefit-risk profile of once-weekly basal insulin icodec, as compared to other marketed basal insulins, exploring the potential impact on patients’ unmet needs, as well as the environmental impact related to injection devices, and future applications in real-world setting.

## 2. Pharmacologic Profile of Insulin Icodec

Icodec is a novel recombinant insulin that has been developed to have an analogue of human insulin with distinct pharmacokinetic (PK) and pharmacodynamic (PD) properties, enabling its use for weekly (instead of daily) administrations in patients affected by diabetes mellitus. 

The once-weekly formulation for icodec was obtained through different technological improvement starting from human insulin, such as substitution of some aminoacidic residues on the human insulin’s backbone peptide, the removal of the last aminoacidic residue on chain B, and conjugation with a miniPEG- γGlu spacer, linked to a 1,20-icosanedioic fatty acid (C20) at Lys in position B29 [19,20]. 

In particular, variations in aminoacidic sequence led to enhanced proteolytic stability, enhanced solubility, reduced insulin receptor affinity and receptor-mediated clearance. The molecular changes reducing the affinity of insulin icodec to the insulin receptor have been demonstrated to be unrelated to changes in the overall efficacy, while only affecting potency, and resulting in a prolonged effect over time, as compared to human insulin [19]. Icodec is therefore a full agonist of human insulin, preserving its mechanism of action [20].

The conjugation with C20 icosanedioic acid led to a reduced receptor-mediated clearance and to an enhanced strong and reversible binding ability with human serum albumin (HSA) [19]. It is known that HSA, with its ligand-binding capacity, is the most important carrier protein for both endogenous and exogenous ligands. In general, when drugs bind HSA, their toxicity and clearance rates are reduced, thus resulting in extended circulation half-lives [21,22]. Nishimura’s in vitro studies demonstrated that in the absence of HSA, icodec had a 50% reduced affinity to insulin receptor, while in the presence of 1.5% of HSA, the affinity of insulin icodec for the insulin receptor was reduced by 97%, compared to human insulin [20], reflecting icodec ability for binding HSA. Findings from in vivo studies confirmed that a very low insulin receptor affinity and robust yet reversible binding to HSA led to an ultra-long pharmacokinetic profile [23].

All these modifications led to the development of the first ultra-long-acting formulation with an ultra-extended half-life equal to 196 h (i.e., approximately 8 days) in humans, so that a single injection was designed to cover basal insulin needs over a week. The median time to reach maximum plasma concentration (T_max_) was 16 h after subcutaneous (SC) injection and the steady state was reached by 3–4 weeks [20]. No clinically significant difference in the overall exposure of insulin icodec was observed across different injection sites (thigh, abdomen, or upper arm), thus indicating a consistent glucose-lowering effect, regardless of the SC injection site [24]. Other marketed long-acting insulins and icodec PK properties are compared in Table 1 [25,26,27]; as observed, icodec has the highest half-life, which is far superior compared to once-daily analogues’ half-lives, T_max_, and steady state achieving time. 

Furthermore, unlike certain long-acting formulations, such as insulin’s glargine and degludec, which rely on the self-assembly of hexamers and multi-hexamers [28,29,30], in the icodec formulation, the hexamer agglomeration does not occur. Indeed, to minimize the potential for local irritation and the risk of immunogenicity associated with an extended presence (one week long) in the subcutaneous tissue, icodec was designed to generate an albumin-bound reservoir, thus providing a slow and steady glucose-lowering effect and reducing the potential risk of immunogenicity [19]. The innovation of icodec relies on absence of subcutaneous depot providing a slow-release mechanism; indeed, this process occurs entirely in the bloodstream, where icodec monomers attach and detach from albumin. The mechanism of protraction is therefore completely independent from the subcutaneous tissue [23]. Furthermore, the inclusion of the icosanedioic fatty acid in the icodec molecule results in icodec exhibiting a binding affinity to albumin approximately seven and a half times greater than the interaction observed between albumin and insulin degludec, and about nine and a half times stronger than that observed with insulin detemir [21].

Recently, an open-label trial, involving 46 individuals with T2DM, was carried out specifically to investigate the steady-state pharmacological properties of this insulin in patients, following its administration at a dose level adjusted to each participant’s individual basal insulin need; the steady state was confirmed to be reached within 3–4 weeks (with a computational model suggesting the possibility of reduction to 2–3 weeks by adding a boosted additional 50% icodec dose at the first administered dose); moreover, the glucose-lowering effect per day during the 1-week dosing interval at the steady state ranged from 12.0% to 16.1% of the total weekly effect, similar to the desirable 14.3% of the daily glucose-lowering effect [31].

With its unique features, icodec represents a pharmacological innovation in the field of basal insulins for the treatment of DM.

## 3. Benefit-Risk Profile of Insulin Icodec, as Compared to Other Basal Insulins

Phase 2 trials, conducted both in insulin-naïve and in previously insulin-treated patients with T2DM, have shown good tolerability and promising glucose-lowering effects of once-weekly insulin icodec [32,33,34]. Treatment with once-weekly insulin icodec, with or without other glucose-lowering medications, was associated with a comparable efficacy to once-daily insulin glargine U100 in insulin-naïve patients, in terms of reductions from baseline glycated hemoglobin (HbA1c) levels [32,33]. Concerning the safety profile, both icodec and glargine U100 groups showed a low incidence rate of combined level 2 (i.e., clinically significant hypoglycemia: blood glucose < 54 mg/dL) or level 3 (i.e., severe hypoglycemia: severe cognitive impairment requiring external assistance for recovery) hypoglycemia. Specifically, the proportion of patients that achieved HbA1c levels <7.0% without developing clinically significant or severe hypoglycemia events was higher in patients receiving insulin icodec rather than those receiving glargine U100, and improved Time in Range (TIR) results were observed both in icodec insulin-naïve patients and in those who switched from other basal insulins [32,33]. Moreover, among previously insulin-treated patients, the addition of a one-time 50% extra dose of icodec at first administration, when switching to icodec, resulted in effective glycemic control without transitory fasting hyperglycemia or increased risk of clinically significant hypoglycemia, when compared to patients who switched to once-daily glargine U100. Interestingly, due to its concentrated formulation, its injection volume was equivalent to that of once-daily insulin glargine U100 [34].

Starting from these findings, the phase 3a ONWARDS clinical trial programme has been developed. It included six distinct RCTs, each specifically developed to address the efficacy and safety of once-weekly insulin icodec in a spectrum of clinical scenarios [35,36,37,38,39,40]. Five RCTs enrolled patients with T2DM and one RCT enrolled patients with type 1 diabetes mellitus (T1DM). Specifically, two of the six RCTs compared insulin icodec vs insulin degludec (a second-generation insulin that currently provides the best benefits and represents the only basal insulin with an ultra-long action [8,10]), both in insulin-naïve patients (ONWARDS 3) and in patients previously treated with basal insulin (ONWARDS 2). Furthermore, in ONWARDS 1 and 5 trials, T2DM insulin-naïve patients were enrolled, while in ONWARDS 4 and 6 patients previously treated with basal-bolus insulin with T2DM (ONWARDS 4) and T1DM (ONWARDS 6) were enrolled.

The primary efficacy endpoint was the change of the glycated hemoglobin level from baseline to the end of each trial in the patient receiving once-weekly insulin icodec vs. once-daily basal insulins, such as degludec, glargine U100, and glargine U300; the major safety endpoints were the rates of level 2 or level 3 hypoglycemia per patient-year of exposure (PYE) in the patient receiving once-weekly insulin icodec vs. once-daily basal insulins.

For insulin-naïve individuals with T2DM, the ONWARDS clinical trials programme suggested a starting dose of 70U weekly. Icodec was administered by subcutaneous injection once-weekly, using a pre-filled pen injector. Titration involved a weekly increase of 20U for icodec and a daily increase of 3U for basal insulins (degludec/glargine) when average fasting blood glucose levels exceeded the target (>130 mg/dL). Conversely, if average fasting blood glucose level was below the target (<80 mg/dL), the weekly dose was decreased by the same amounts for both icodec and other insulins. For non-insulin-naïve patients, the initial dose was set at 7× the previous daily basal insulin dose. For the first injection only, an additional one-time 50% of icodec dose was administered. Titration was performed, as for insulin-naïve patients, starting from week 2. Dose titrations, including adjustments related to potential risks (e.g., hypoglycemia), were made by the investigator overseeing the study participant [41].

**Figure 1:**
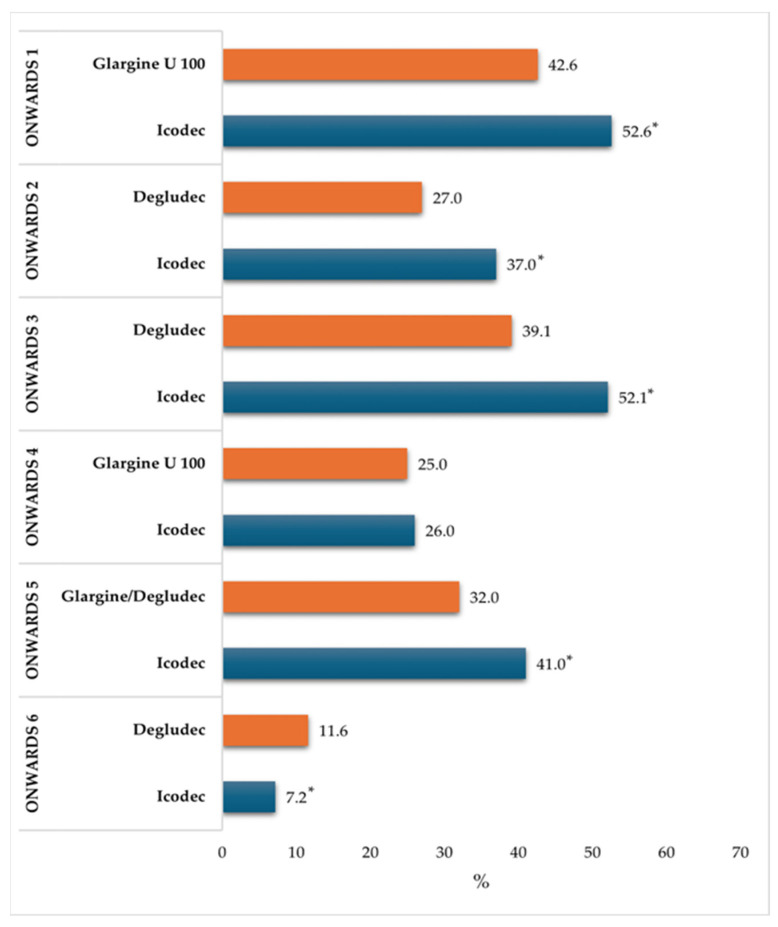
Rate of patients that achieved HbA1c levels <7.0% without developing lv. 2^a^ or lv. 3^b^ hypoglycemic events in the ONWARDS clinical trial program. Abbreviations: Lv. = Level; HbA1c = glycated hemoglobin. ^a^ Clinically significant hypoglycemia: blood glucose level <54 mg/dL (<3.0 mmol/L) confirmed by blood glycosometer; ^b^ Severe hypoglycemia: hypoglycemia associated with severe cognitive impairment requiring assistance for recovery; * Statistically significant difference observed between icodec and comparator.

Features, along with primary efficacy and safety outcomes of the ONWARDS trial programme, are described below and summarized in Table 2 and Figure 1.

ONWARDS 1 (NCT04460885) was a 78-week, randomized, open-label, treat-target, phase 3a trial that involved a total of 984 insulin-naïve patients randomized to receive either once-weekly icodec or once-daily glargine U100. Participants were allowed to continue previous non-insulin glucose-lowering medications, except for sulphonylureas (SUs) and glinides, which were discontinued at randomization. The impact of icodec was assessed after a 52-week primary phase, followed by a 26-week extension phase aimed at evaluating efficacy and long-term safety. The mean reduction in the glycated hemoglobin level at 52 weeks was greater with icodec (8.50% to 6.93%; mean change −1.55%) than with glargine U100 (8.44% to 7.12%; mean change −1.35%); the estimated treatment difference (ETD = −0.19%; 95%CI: −0.36 to −0.03) confirmed the non-inferiority (*p* < 0.001) and superiority (*p* = 0.02) of icodec compared to glargine. In the last 5 weeks (i.e., week 48–52) of the trial, participants receiving icodec were longer in the target glycemic range (70–180 mg/dL) than those receiving glargine U100 (71.9% vs. 66.9%; ETD = 4.27%; 95% CI: 1.92 to 6.62; *p* < 0.001). Rates of combined level 2 or 3 hypoglycemia were 0.30 events per PYE, with icodec and 0.16 events per PYE with glargine U100 at week 52 (estimated rate ratio = 1.64; 95% CI: 0.98 to 2.75) and 0.30 and 0.16 events per PYE, respectively, at week 83 (estimated rate ratio = 1.63; 95% CI: 1.02 to 2.61). The rate of patients that achieved an HbA1c <7.0% without developing clinically significant or severe hypoglycemic events at week 52 were significantly higher in patients receiving insulin icodec rather than the control (52.6% vs. 42.6%; estimated odds ratio = 1.49; 95%CI: 1.15 to 1.94). No new safety signals were identified, and incidences of adverse events were similar in the two groups [35].

ONWARDS 2 (NCT04770532) was a randomized, open-label, treat-to-target, 26-week trial including 526 T2DM patients previously treated with basal insulin, with or without non-insulin anti-diabetic treatment (except SUs or glinides), who switched to once-weekly insulin icodec versus those who switched to once-daily insulin degludec. The mean reduction in the glycated hemoglobin level at 26 weeks was greater with icodec (8.17% to 7.20%; mean change: −0.93%) than with degludec (8.10% to 7.42%; mean change: −0.71%); the ETD (−0.22%; 95% CI: −0.37 to −0.08) confirmed the non-inferiority (*p* < 0.001) and superiority (*p* = 0.0028) of icodec compared to degludec. In the last 5 weeks (i.e., week 22–26) of the trial, participants receiving icodec were longer in the target glycemic range (70–180 mg/dL) than those receiving degludec (63.1% vs. 59.5%; ETD = 2.41%; 95% CI: −0.84 to 5.65; *p* = 0.15). Rates of combined level 2 or 3 hypoglycemia were 0.73 events per PYE with icodec and 0.27 events per PYE with degludec at week 26 (estimated rate ratio = 1.93; 95% CI: 0.93 to 4.02). The rate of patients that achieved an HbA1c <7.0% without developing clinically significant or severe hypoglycemia events at week 26 were significantly higher in patients receiving insulin icodec rather than the control (37% vs. 27%; estimated odds ratio = 1.59; 95%CI: 1.07 to 2.36). No new safety issues were identified between the two groups in the trial [36].

ONWARDS 3 (NCT04795531) was a double-blind, treat-to-target, 26-week trial that involved a total of 588 insulin-naïve patients randomized to receive either once-weekly icodec or once-daily insulin degludec. The mean reduction in the glycated hemoglobin level at 26 weeks was greater with icodec (8.6% to 7.0%; mean change: −1.6%) than with degludec (8.5% to 7.2%; mean change: −1.4%); the ETD (−0.2%; 95% CI: −0.3 to −0.1) confirmed the non-inferiority (*p* < 0.001) and superiority (*p* = 0.002). Rates of combined level 2 or 3 hypoglycemia were statistically higher for icodec (0.35 events per PYE) than for degludec (0.12 events per PYE with) at week 26 (estimated rate ratio = 3.12; 95% CI: 1.30 to 7.51), while statistical significance was lost at week 31 (estimated rate ratio = 1.82; 95% CI: 0.87 to 3.80). The rate of patients that achieved an HbA1c < 7.0% without developing clinically significant or severe hypoglycemia events at week 26 were higher in patients receiving insulin icodec rather than the control (52.1% vs. 39.1%). Overall safety profile was comparable to insulin degludec [37]. 

ONWARDS 4 (NCT04880850) was a 26-week randomized, open-label, treat-to-target trial that compared once-weekly insulin icodec to once-daily insulin glargine U100, in combination with mealtime aspart (2–4 times per day) in 582 people with type 2 diabetes, previously receiving basal-bolus therapy. The mean reduction in the glycated hemoglobin level at 26 weeks was greater with icodec (8.29% to 7.14%, mean change −1.16%) than with glargine U100 (8.31% to 7.12%; mean change −1.18%); the ETD (0.02%; 95% CI: −0.11 to 0.15; *p* < 0.0001) confirmed the noninferiority of icodec compared to glargine. In the last 5 weeks (i.e., week 22–26) of the trial, participants receiving icodec spent a non-significantly greater time in the target glycemic range (70–180 mg/dL) than those receiving glargine (66.9% vs. 66.4%; ETD = 0.29%; 95% CI: −2.52 to 3.09; *p* = 0.84). Rates of combined level 2 or 3 hypoglycemia were similar for icodec and glargine groups: 5.64 events per PYE with icodec and 5.62 events per PYE with glargine at week 26, respectively (estimated rate ratio = 0.99; 95% CI: 0.73 to 1.33). As expected, the number of events per PYE was higher in this trial, where patients received basal-bolus insulin treatment, compared to other trials. The rate of patients that achieved an HbA1c < 7.0% without developing clinically significant or severe hypoglycemia events at week 26 did not differ in patients receiving insulin icodec rather than the control (26% vs. 25%; estimated odds ratio = 1.07; 95%CI: 0.73 to 1.55). No new safety issues were identified between the two groups in the trial [38].

ONWARDS 5 (NCT04760626) was a 52-week, randomized, open-label, multinational, phase 3a trial with real-world elements, that involved a total of 1085 insulin-naïve patients randomized to receive either once-weekly icodec with a dosing guide app or once-daily insulin degludec or glargine U100 or glargine U300. The mean reduction in the glycated hemoglobin level at 52 weeks was greater with icodec (8.96% to 7.24% mean change: −1.68%) than with other basal insulins (8.88% to 7.61% mean change: −1.31%); the ETD (−0.38%; 95% CI: −0.66 to −0.09) confirmed the non-inferiority (*p* < 0.001) and superiority (*p* = 0.009) of icodec compared to once-daily basal insulin analogues. Rates of combined level 2 or 3 hypoglycemia were similar and low for icodec and other basal insulins groups: 0.19 events per PYE with icodec and 0.14 events per PYE with other basal insulins at week 52, respectively (estimated rate ratio = 1.17; 95% CI: 0.73 to 1.86). The rate of patients that achieved an HbA1c < 7.0% without developing clinically significant or severe hypoglycemia events at week 52 were significantly higher in patients receiving insulin icodec rather than the control (41% vs. 32%; estimated odds ratio = 1.47; 95%CI: 1.13 to 1.92). No new safety issues were identified between the two groups in the trial [39].

ONWARDS 6 (NCT04848480) was a 52-week (26-week main phase plus a 26-week safety extension phase), open-label, randomised, treat-to-target, phase 3a trial that enrolled a total of 582 T1DM patients, previously receiving basal-bolus therapy, randomized to receive either once-weekly icodec or once-daily insulin degludec, in combination with mealtime aspart (≥2 times per day). Insulin icodec successfully met its primary endpoint of reducing glycated hemoglobin. At week 26, the mean glycated hemoglobin reduction was similar for insulin icodec (7.59% to 7.15%; mean change: −0.47%) and for degludec (7.63% to 7.10%; mean change: −0.51%); the ETD (0.05%; 95% CI: −0.13 to 0.23) confirmed non-inferiority of icodec compared to degludec (*p*-value = 0.0065). Rates of combined level 2 or 3 hypoglycemia were significantly higher for once-weekly insulin icodec rather than once-daily degludec: 19.93 events per PYE vs. 10.37 events per PYE, respectively (estimated rate ratio = 1.9; 95% CI: 1.5 to 2.3). The rate of patients that achieved an HbA1c <7.0% without developing clinically significant or severe hypoglycemia events at week 52 were significantly lower in patients receiving insulin icodec rather than the control (7.2% vs. 11.6%; estimated odds ratio = 0.59; 95%CI: 0.37 to 0.95) [40].

As demonstrated by ONWARDS findings, insulin icodec showed a comparable clinical efficacy profile with basal insulin analogues, in both T1DM and T2DM patients, regardless of whether patients had previously been treated with basal insulin or not. In particular, a superior clinical efficacy of insulin icodec, compared to the standard basal insulin treatment currently available, was demonstrated in ONWARDS 1, 2, 3, and 5, where enrolled patients were on basal insulin/oral antidiabetic treatment.

The transition from daily to weekly intake represents an enormous advantage for T2DM patients, who are often elderly people with comorbidities and in polytherapy. Such important and convincing results from the clinical trials, in terms of efficacy and safety, along with the innovative pharmacological features, place some important expectations on icodec. In particular, it is expected that icodec would impact positively on crucial factors in the diabetes management, such as compliance with treatment and quality of life, and also on other elements, such as healthcare costs and environmental impact, which are very relevant aspects in today’s society. 

## 4. Potential Impact and Innovative Aspects of Once-Weekly Basal Insulin Administration

### 4.1. Unmet Needs and Compliance

Adherence to antidiabetic therapy is one of the main factors that contribute to good control of diabetes over time and to a lower risk of developing disease-related complications. Furthermore, a good adherence to treatment may positively impact on decreasing the number of emergency department visits, hospitalizations, and medical care costs [42,43,44,45]. However, a significant proportion of individuals with diabetes struggle to consistently follow their prescribed insulin regimens, interrupting or discontinuing insulin treatment shortly after initiation [46,47,48]. As a consequence, more than 50% of people with T2DM receiving basal insulin fail to achieve glycemic goals [49]. The reason can be attributed to social factors, low socioeconomic status, complexity of diabetes treatment, impact of insulin therapy on daily life, fear of injection, pain, or side effects that collectively influence patient adherence to therapy [50]. 

Concerning complexity of diabetes treatment and impact of insulin therapy on daily life, numerous studies exploring patients’ perceptions on insulin treatment were conducted [51,52]. A survey published in 2012 showed that the number of daily injections and the need to receive insulin injections at prescribed times every day were the two most reported issues for patients with insulin treatment. Furthermore, patient agreement was strongest for the wish that good blood glucose control with insulin should not require injections every day (92.5%) and that insulin administrations would fit daily life changes (81.4%) [51]. A more recent survey, published in 2024, was conducted to specifically explore patients’ and providers’ preferences on the use of once-weekly basal insulin. The results of this survey showed that the vast majority of the sample of patients with T2DM (91%) and health care practitioners (89%) would prefer a once-weekly insulin rather than another type of basal insulin. Furthermore, T2DM patients already treated with basal insulin expressed greater confidence in the potential of a once-weekly insulin regimen to better manage their blood sugar levels [53]. 

The burden of daily insulin injection negatively affects the quality of life (QoL) of diabetic patients and for this reason simplified regimens may be generally preferred [52]. Indeed, higher rates of insulin non-adherence are in general observed among those patients who perceived insulin therapy as restrictive and inflexible, particularly regarding the timing of injections [54,55,56]. 

Moreover, non-compliance with medication in general imposes a substantial financial burden on healthcare systems [57], and diabetic disease is not exempt from this impact [44,58,59,60,61]. A retrospective cohort study conducted in 2020 on 13,296 diabetic patients receiving basal insulin treatment showed that the mean annual direct healthcare cost per patient was significantly lower for adherent (defined as proportion of days covered (PDC) ≥80%) patients, compared to non-adherent patients (30,127$ vs. 37,049$). In particular, the savings were mainly attributable to outpatient (13,839$ vs. 18,988$) and acute care (6181$ vs. 10,054$) costs, while costs related to medication were higher for adherent patients, compared to non-adherent patients (11,606$ vs. 7480$) [58]. A cohort study involving more than 740,000 patients with T2DM showed that non-adherence to treatment (defined as medication possession ratio (MPR) < 80%) was associated with an increase of 41% of the total inpatient cost [62]. Another issue is the burden on caregivers, especially for elderly non-self-sufficient diabetic patients; thanks to the steady glucose profiles maintained over weeks, a reduced need for frequent adjustments is expected with once-weekly formulations, providing advantages for patients and their caregivers [63].

The Italian regulatory agency recognizes the need and potential for therapies aimed at improving adherence to treatment, such as those reducing the complexity of the treatment, this being one of the main reasons causing poor adherence [64]. 

In summary, improved adherence achievable with weekly insulin therapy not only offers potential clinical and psychophysical advantages, but also could lead to significant economic benefits, as demonstrated by the lower annual per-patient costs, particularly in outpatient and inpatient care. It is crucial for clinicians to understand emotions, anxieties, and difficulties of patients starting an insulin treatment. Clinicians should also identify the factors that motivate patients to persist with the therapy, and the factors that lead patients to discontinue or interrupt insulin treatment.

In this context, implementing an insulin regimen that involves less administration, like what icodec might provide, could offer greater comfort and convenience to patients, thus discouraging them from missing doses.

### 4.2. Quality of Life

#### 4.2.1. The Analogy with GLP1-RA: Once-Daily vs. Once-Weekly Formulation

The impact of once-weekly subcutaneous administration of a glucose lowering medication on QoL of diabetic patients was recently experienced with glucagon-like peptide-1 receptor agonists (GLP1-RA). GLP1-RA are frequently advised as an effective therapy for T2DM patients when oral medications alone fail to achieve therapeutic and glycemic goals [4]. The first GLP1-RA analogue was exenatide, approved by FDA in 2005, which was administered twice a day. In the following years, different GLP1-RA analogues, comparable in terms of tolerability and efficacy, were developed both as once-daily and once-weekly formulations [65,66].

Several studies conducted in real-world setting showed that adherence and persistence to GLP1-RA were better in patients taking once-weekly formulation, compared to once/twice daily formulations, with a probability of achieving an higher adherence ranging from 11% to 78% [67,68,69,70,71]. Results from these studies might have multiple explanations: firstly, once-weekly administration might be convenient for patients with busy lifestyles; by simplifying the treatment schedule and reducing the frequency of injections, there is a reduced effort for patients to remember and adhere to a daily medication regimen, thus resulting in an enhanced overall compliance with the prescribed therapeutic plan. Secondly, some patients may have needle phobia, an anxiety related to injections or injection site fatigue because of frequent administrations. In this context, a reduced number of injections can reduce both the psychological and physical burden associated with daily administration. Moreover, other evidences showed that patients that have previously been treated with both once-daily and once-weekly formulations strongly preferred once-weekly administration, in terms of convenience/flexibility, global treatment satisfaction and device ease of use [72,73]. 

The improved adherence and persistence observed in patients using once-weekly GLP1-RA formulations, compared to once- or twice-daily regimens, and the patients’ positive treatment perceptions, combined with the analogies in terms of administration route and dosing frequency, suggest that a similar phenomenon is likely to be observed in the case of insulin therapy. The transition from daily to weekly intake could represent an important turning point in diabetes management, importantly impacting on the quality of life of patients.

#### 4.2.2. T2DM Patients’ Treatment Satisfaction in the ONWARDS Trials Programme

Clinical trials ONWARDS 2 and 5 investigated the general patient satisfaction with insulin icodec treatment, compared with degludec or degludec/glargine, respectively, in patients with T2DM, using some validated tools that measured treatment satisfaction and patient compliance [36,39]. Treatment satisfaction differences between icodec and comparator were evaluated through the Diabetes Treatment Satisfaction Score Questionnaire (DTSQ) [74] in both ONWARDS 2 and 5 RCTs, while compliance domain score differences were assessed through the Treatment Related Impact Measure for Diabetes (TRIM-D) [75] in ONWARDS 5. DTSQ assessed patient satisfaction, perceived control of blood sugar levels, flexibility of treatment, and likelihood of recommending the treatment to others, while TRIM-D measured the emotional burden, the perceived health status, and the treatment-related hassles associated with daily management. In both ONWARDS 2 and 5 RCTs, the mean change in DTSQ score from baseline to week 26 (ONWARDS 2) or week 52 (ONWARDS 5) was greater for icodec (4.22 and 4.68, respectively) vs. other basal insulins (2.96 and 3.90, respectively), with an ETD equal to 1.25 (95% CI: 0.41 to 2.10; *p* = 0.0035) and to 0.78 (95% CI: 0.10 to 1.47; *p* = not reported), respectively. Furthermore, the TRIM-D score was greater for icodec (90.42 and 87.37) vs. other basal insulins, with an ETD equal to 3.04 (95% CI: 1.28 to 4.81) at week 52 in ONWARDS 5 RCT [36,39]. 

These findings, in line with data on patients receiving once-daily vs. once-weekly GLP1-RA [72,73], showed that patients with T2DM preferred once-weekly insulin formulation, compared to once-daily formulation.

Icodec has the potential to simplify the treatment of diabetes that requires insulin therapy, eliminating the discomfort of the daily injection for patients, thus hopefully increasing adherence to treatment.

### 4.3. Environmental Impact of Devices

The environmental impact related to drugs manufacturing represents a critical issue for the entire society. Regulators are seriously taking into account this aspect by implementing the environmental impact in the Health Technology Assessment (HTA) evaluation of new drugs [76,77].

Concerning specifically insulin injectors, these may have an impact on the environment, both during manufacturing and in the after-use disposal. Pharmaceutical industries are significantly impacted by carbon dioxide (CO_2_) emissions, with medical devices being a major contributor. As a result, prioritizing efforts to reduce the climate footprint of these devices is essential in the overall decarbonization process of the healthcare sector [78]. Single-use devices are widely used, especially in high-income countries [79], due to their advantages in terms of reduction of procedures’ complexity and inventory costs [80]; they also can reduce the pain associated with the administration [81], and are perceived as capable of reducing the risk of health-care infections, compared to reusable devices [82]. Nevertheless, these benefits are accompanied by the drawback of adding several grams of plastic waste, with a resulting environmental and public health damage [78]. Diabetes care product itself is around only 10% of the total weight and volume that was delivered, while the remaining 90% is mainly plastic, metal, and paper or cardboard for the packaging [83]. To have an idea of the amount of plastic waste generated from pen-injector diabetes care devices, Novo Nordisk stated that more than 550 million pen devices are yearly developed and delivered worldwide, each one containing around 77% of plastic that cannot be thrown into the plastic recycling bin [84]. Moreover, insulin injectors generate biomedical waste, including needles and cartridges. Improper disposal of these materials can be hazardous to the environment and public health, unless appropriately managed [78].

Sensitivity towards environmental protection should be a priority for drug manufacturers that should aim to promote solutions to reduce CO_2_ emissions and improve sustainability. In this context, reducing the frequency of insulin administrations from daily to once per week may result in a remarkable reduction of the yearly production of pen injectors, which could have a significant environmental impact by reducing CO_2_ emissions and plastic waste. 

## 5. Future Applications in Real-World Setting: Place in Therapy

Insulin therapy plays a crucial role in managing both T1DM and T2DM. Many individuals with T2DM eventually require and benefit from insulin therapy, especially those with uncontrolled diabetes or with complications; basal insulin can be administered in monotherapy or in addition to oral antidiabetic drugs/non-insulin injectables, as stated in the latest international guidelines [4,5]. Real-world data show that insulin is one of the most prescribed antidiabetic drugs; in particular, among antidiabetic drug users, insulin use ranges between 10–30% [85,86,87,88], while in newly diagnosed T2DM patients insulin use as a starting treatment ranges between 5–12% [89,90,91]. By 2030, the global estimate for the number of patients receiving insulin is projected to exceed 37 million, whereas in 2018 it was around 30 million [92]. 

In this context, icodec is a novel basal insulin that has an ultra-long half-life that guarantees a once-weekly administration. Non-inferiority in terms of efficacy, compared to standard basal insulin treatment, was confirmed by 6 phase III RCTs that involved more than 4000 patients affected by diabetes mellitus; furthermore, superiority of insulin icodec was confirmed in insulin-naïve T2DM patients where safety profile was demonstrated to be comparable to that of standard basal insulins without additional risks. 

Given its pioneering pharmacological features, along with its proven positive benefit-risk profile, and the relevant epidemiological context, icodec is expected to significantly impact diabetes management, especially in T2DM insulin-naïve patients, ensuring healthcare professionals have a new reliable and efficient therapeutic option for those patients needing an insulin treatment. In particular, icodec, with its innovative once-weekly administration, has the potential to address the issue of complicated and time-consuming insulin regimens experienced by patients, thus hopefully increasing overall adherence to treatment [93], achieving the glycemic targets. Furthermore, using once-weekly icodec, patients with diabetes will switch from 365 to just 52 injections per year. Lastly, by providing a viable and cost-effective solution, icodec will hopefully determine an overall cost saving for health care providers involved in the management of diabetic disease, aligning with the imperative to optimize resource utilization and enhance overall healthcare efficiency.

## 6. Conclusions

Insulin icodec, with its unique molecular and pharmacological features, provides an innovative once-weekly treatment option for the treatment of diabetes mellitus, being one of the most important therapeutic innovations in the field. The ONWARDS trial programme confirmed that icodec efficacy profile was non-inferior or even superior (i.e., in T2DM insulin-naïve patients) to the currently available basal insulins, with similar glycemic control and comparable rates of hypoglycemic events, especially for T2DM patients. The therapeutic added value of insulin icodec relies on the potential to improve patients’ adherence to insulin treatment, thus leading to a better achievement of therapeutic goals and influencing positively both patients’ treatment satisfaction and overall QoL, reducing the burden for patients and caregivers. Finally, icodec could also impact both on the overall healthcare costs related to non-adherent diabetic patients’ management, and on the environment by reducing plastic waste and CO_2_ emissions related to pen-injector manufacturing. Future real-world studies may be helpful to explore the uptake and to confirm the long-term benefit-risk profile of insulin icodec in clinical practice, as well as the impact of this drug on the patient’s adherence to treatment in the real-world setting.

A true epochal change is yet to come in diabetes management and both patients and the healthcare system will be the greatest beneficiaries.

## Figures and Tables

**Table 1:** Pharmacokinetic profile of different basal insulin formulations.

Insulin, Type	T ½ (h)	Tmax (h)	Steady State (Day)
NPH Insulin	4.4	8	N/A
Insulin detemir	7	8	2–4
Glargine U100	12.1	8–12	2–4
Glargine U300	19.0	12–16	3–5
Degludec	25.4	10–12	2–3
Icodec	196	16	21–28

Abbreviations: h = hours; N/A = not available; NPH = Neutral Protamine Hagedorn.

**Table 2:** RCT characteristics and main findings on efficacy and safety outcomes from the ONWARDS clinical trial program.

Trial Information								Efficacy Outcomes				Safety Outcomes	
Trial	Countries	Phase	Duration (Weeks)	DM Type	Insulin Treatment Experience	N. of Enrolled Patients	Insulin Treatment	Mean Baseline HbA1c (%)	End of Trial Mean HbA1c (%)	HbA1c Mean Change (%)	ETD (S and NI *p*-Values)	Rate of Combined Level 2 ^a^ or level 3 ^b^ Hypoglycemia (Events per PYE)	ERR (CI 95%; *p*-Value)
ONWARDS 1	Croatia, India, Israel, Italy, Japan, Mexico, Poland, Puerto Rico, Russia, Slovakia, Spain, UK, USA	III	52 + 26	Type 2	Insulin-naïve	492	Icodec	8.50	6.93	−1.55	−0.19 (S: *p* = 0.02, NI: *p* < 0.001)	0.30	1.64 (0.98–2.75; *p* = NR)
						492	Glargine U100	8.44	7.12	−1.35		0.16	
ONWARDS 2	Bulgaria, Germany, Japan, Korea, Poland, Portugal, South Africa, Ukraine, USA	III	26	Type 2	Previously treated with basal insulin	263	Icodec	8.17	7.20	−0.93	−0.22 (S: *p* = 0.0028 NI: *p* < 0.001)	0.73	1.93 (0.93–4.02; *p* = 0.078)
						263	Degludec	8.10	7.42	−0.71		0.27	
ONWARDS 3	Argentina, Austria, Brazil, Canada, China, Czechia, Denmark, France, Mexico, Puerto Rico, Taiwan, USA	III	26	Type 2	Insulin-naïve	294	Icodec	8.60	7.00	−1.60	−0.2 (S: *p* = 0.002 NI: *p* < 0.001)	0.35	1.82 (0.87–3.80; *p* = 0.11)
						294	Degludec	8.50	7.20	−1.40		0.12	
ONWARDS 4	Belgium, India, Italy, Japan, Mexico, Netherlands, Romania, Russia, USA	III	26	Type 2	Previously treated with basal-bolus insulin	291	Icodec +2–4 times/day aspart	8.29	7.14	−1.16	0.02 (S: *p* = NR, NI *p* < 0.001)	5.64	0.99 (0.732–1.33; *p* = 0.93)
						291	Glargine U100 +2–4 times/day aspart	8.31	7.12	−1.18		5.62	
ONWARDS 5	Canada, Germany, Greece, Hungary, Poland, Puerto Rico, Turkey, USA	III	52	Type 2	Insulin-naïve	542	Icodec	8.96	7.24	−1.68	−0.38 (S: *p* = 0.009, NI: *p* < 0.001)	0.19	1.17 (0.73–1.86; *p* = NR)
						543	Glargine U100/Glargine U300 /Degludec	8.88	7.61	−1.31		0.14	
ONWARDS 6	Austria, Canada, Germany, India, Italy, Japan, Netherlands, Russian Federation, Spain, Turkey, UK, USA	III	52	Type 1	Previously treated with basal-bolus insulin	290	Icodec +≥2 times/day aspart	7.59	7.15	-0.47	0.05 (S: *p* = NR, NI: *p* = 0.007)	19.93	1.9 (1.5–2.3; *p* < 0.001)
						292	Degludec +≥2 times/day aspart	7.63	7.10	-0.51		10.37	

Abbreviations: DM = diabetes mellitus; HbA1c = glycated hemoglobin; ETD = estimated treatment difference; ERR = estimated rate ratio; S = superiority; NI = non-inferiority; NS = not statistically significant; PYE = patient-year exposure; NR = not reported; CI= confidence interval; UK = United Kingdom; USA = United States of America. ^a^ Clinically significant hypoglycemia: blood glucose level <54 mg/dL (<3.0 mmol/L) confirmed by blood glycosometer; ^b^ Severe hypoglycemia: hypoglycemia associated with severe cognitive impairment requiring assistance for recovery
